# FLT3 is implicated in cytarabine transport by human equilibrative nucleoside transporter 1 in pediatric acute leukemia

**DOI:** 10.18632/oncotarget.10448

**Published:** 2016-07-06

**Authors:** Albert Català, Marçal Pastor-Anglada, Liska Caviedes-Cárdenas, Roberta Malatesta, Susana Rives, Nerea Vega-García, Mireia Camós, Paula Fernández-Calotti

**Affiliations:** ^1^ Pediatric Hematology and Oncology Department, Hospital Sant Joan de Déu, University of Barcelona, Esplugues de Llobregat, Barcelona, Spain; ^2^ National Biomedical Research Institute on Rare Diseases (CIBER ER), Instituto de Salud Carlos III, Madrid, Spain; ^3^ Department of Biochemistry and Molecular Biology, University of Barcelona, Institute of Biomedicine (IBUB), Barcelona, Spain; ^4^ Oncology Program, National Biomedical Research Institute of Liver and Gastrointestinal Diseases (CIBER EHD), Instituto de Salud Carlos III, Madrid, Spain; ^5^ Hematology Laboratory, Hospital Sant Joan de Déu, University of Barcelona, Esplugues de Llobregat, Barcelona, Spain; ^6^ Institut de Recerca Pediàtrica Hospital Sant Joan de Déu (IRP-HSJD), Esplugues de Llobregat, Barcelona, Spain

**Keywords:** FLT3, Cytarabine, hENT1, nucleoside transporters, pediatric acute leukemia

## Abstract

*FLT3* abnormalities are negative prognostic markers in acute leukemia. Infant leukemias are a subgroup with frequent *MLL (KMT2A)* rearrangements, *FLT3* overexpression and high sensitivity to cytarabine, but dismal prognosis. Cytarabine is transported into cells by Human Equilibrative Nucleoside Transporter-1 (hENT1, SLC29A1), but the mechanisms that regulate hENT1 in acute leukemia have been scarcely studied.

We explored the expression and functional link between FLT3 and main cytarabine transporters in 50 pediatric patients diagnosed with acute lymphoblastic leukemia and *MLL* rearrangement (ALL-MLL+) and other subtypes of leukemia, and in leukemia cell lines.

A significant positive correlation was found between *FLT3* and *hENT1* expression in patients. Cytarabine uptake into cells was mediated mainly by hENT1, hENT2 and hCNT1. hENT1-mediated uptake of cytarabine was transiently abolished by the FLT3 inhibitor PKC412, and this effect was associated with decreased *hENT1* mRNA and protein levels. Noticeably, the cytotoxicity of cytarabine was lower when cells were first exposed to FLT3 inhibitors (PKC412 or AC220), probably due to decreased hENT1 activity, but we observed a higher cytotoxic effect if FLT3 inhibitors were administered after cytarabine.

FLT3 regulates hENT1 activity and thereby affects cytarabine cytotoxicity. The sequence of administration of cytarabine and FLT3 inhibitors is important to maintain their efficacy.

## INTRODUCTION

Acute leukemia is the most frequent cancer in children. Although a remarkable improvement in survival of children with acute leukemia has been achieved, some patients still have a poor outcome. For these high-risk patients it is still necessary to find new biomarkers that may guide therapy or serve themselves as a target for new therapies. Among high-risk cases, infant leukemias (those diagnosed at age < 1 year) are a special subgroup with distinctive clinical and biological features, including frequent *MLL* (*Mixed Lineage Leukemia,* also known as *KMT2A*) rearrangements, high *FLT3* (*FMS-like tyrosine kinase 3*) expression and high sensitivity to the nucleoside analogue cytarabine (Ara-C), but a dismal prognosis [1–6].

FLT3 is a tyrosine-kinase receptor with a key role in hematopoiesis. FLT3 mutations and overexpression have emerged as negative prognostic biomarkers in acute lymphoblastic leukemia (ALL) and acute myeloblastic leukemia (AML) [3, 7–10]. FLT3 is expressed in the majority of ALL and AML cases, but the highest levels have been observed in ALL with *MLL* gene rearrangements (ALL-MLL+) and hyperdiploid ALL (51–67 chromosomes, HeH) [6, 10]. FLT3 overexpression correlates with unfavorable prognosis in adult AML cases as well as in infant leukemias [8]. The overexpression of FLT3, even in the absence of activating mutations, causes phosphorylation of the receptor, resulting in a constitutively active form of FLT3 in a ligand-independent manner [3, 5, 8, 9, 11]. Internal tandem duplication of the *FLT3* gene (FLT3-ITD) constitutively activates the receptor and confers a negative prognostic impact in both adult and pediatric AML cases, especially when a high mutant to wild-type allelic ratio is observed [3, 8, 12, 13].

Ara-C is a nucleoside analog that requires membrane transport proteins of the human nucleoside transporters (hNT) gene families *SLC28* and *SLC29* to be internalized into target cells. *SLC28* encode human Concentrative Nucleoside Transporters (hCNT) whereas *SLC29* encode human Equilibrative Nucleoside Transporter (hENT) proteins. Despite important advances in the knowledge of the tissue distribution and pharmacology of hNT proteins, there is still a limited understanding of the mechanisms that regulate their expression and activity [14]. Ara-C is known to be transported across the cell membrane mainly by Human Equilibrative Nucleoside Transporter 1 (hENT1, SLC29A1) [14, 15]. Once inside the cell, Ara-C undergoes metabolic activation, and the first and rate-limiting step in this process is its phosphorylation by deoxycitidine kinase (DCK), necessary to finally exert its cytotoxic action [16].

Uptake into cells is a key step in the bioavailability and pharmacological action of nucleoside analogues [16] and, accordingly, several studies have established a correlation between high *hENT1* expression levels, drug sensitivity and outcome [14]. In this regard, elevated *hENT1* expression has been reported to facilitate the high Ara-C sensitivity of infant ALL-MLL+, and a strong correlation between *hENT1* levels and Ara-C sensitivity has been reported in those cases, as well as in adult AML patients [17–19]. On the contrary, low hENT1 expression levels have been related to Ara-C resistance in childhood AML. Overall, these data are in line with the evidence that *in vitro* Ara-C sensitivity in childhood and adult AML is dependent upon hENT1 [19].

In this setting, given that ALL-MLL+ cases present both high levels of FLT3 and high sensitivity to Ara-C, we hypothesized that FLT3 is a suitable candidate to modulate hNT expression and activity, thereby contributing to cell chemosensitivity. To address this issue, we analyzed the relationship between *FLT3* expression levels and mutations and the expression and activity of different hNT and Ara-C metabolizing enzymes in different cell lines and in a series of ALL and AML pediatric patients.

## RESULTS

### *FLT3* is highly expressed in pediatric ALL-MLL+ patients

*FLT3* mRNA expression was quantified by RQ-PCR in 50 patients and 3 cell lines (MV4-11, SEM, K562) and normalized against mRNA from commercial bone marrow CD34+ cells. The expression of *FLT3* was heterogeneous, with a median (arbitrary units) of 4.35 (0.09–4470). Among the different cytogenetic subgroups, the highest *FLT3* levels were observed in ALL-MLL+ patients; none of them had FLT3-ITD (data not shown). We found no significant differences between *FLT3* levels and gender, CNS status or white blood cell count.

### Positive correlation between hENT1 and FLT3 mRNA expression in pediatric leukemia samples

**Figure 1:**
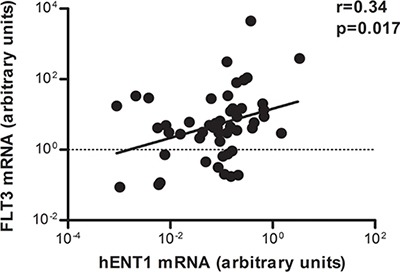
Correlation between *hENT1* and *FLT3* mRNA expression in pediatric leukemia samples Relative *hENT1* mRNA levels of cells from 50 pediatric patients with acute leukemia were plotted against the levels of *FLT3* mRNA in the same samples. Correlation coefficient is shown in the figure.

The mRNA amounts of the main nucleoside transporters (NT) and intracellular metabolizing enzymes (ME), *hENT1, hENT2 (SLC29A2), hCNT1 (SLC28A1), hCNT2 (SLC28A2), hCNT3 (SLC28A3), DCK* and *cN-II (NT5C2),* were measured in 50 pediatric leukemia cases by RQ-PCR. When correlating *FLT3* mRNA levels with all these genes, noticeably, despite the high interpatient variability, a positive correlation was found between *FLT3* and *hENT1* mRNA levels (Figure 1). Based upon these observations we could hypothesize that samples with high *FLT3* mRNA levels would show high Ara-C uptake and probably, a better response to therapy. On the other hand, no significant differences were found between *hENT1, hENT2, hCNT1, hCNT2*, and *hCNT3, DCK* and *cN-II* mRNA expression levels and age, gender, CNS status and WBC count. Nevertheless, a positive correlation between *FLT3* and *DCK* was also observed (Supplementary Figure S1).

Although our study was not designed to analyze NT or ME expression patterns in acute leukemia molecular subgroups, we also found significantly higher *hENT1* levels in AML-MLL+ and lower *DCK* and *cN-II* levels in AML cases and T-ALL samples.

### Characterization of the mRNA expression and associated biological function of nucleoside transporters in leukemic cell lines

As nucleoside transporter expression in pediatric leukemic cells is not well known we analyzed the mRNA expression levels of the most relevant NT that could potentially be implicated in Ara-C uptake, *hENT1, hENT2, hCNT1* and *hCNT3,* in MV4-11, SEM and K562 leukemic cell lines. The former two cell lines originated from childhood leukemia. We observed that *hENT1, hENT2* and *hCNT1* expression is retained in the three cell lines, whereas barely detectable *hCNT3* mRNA was found in SEM and K562 cell lines (Supplementary Table S1).

**Figure 2:**
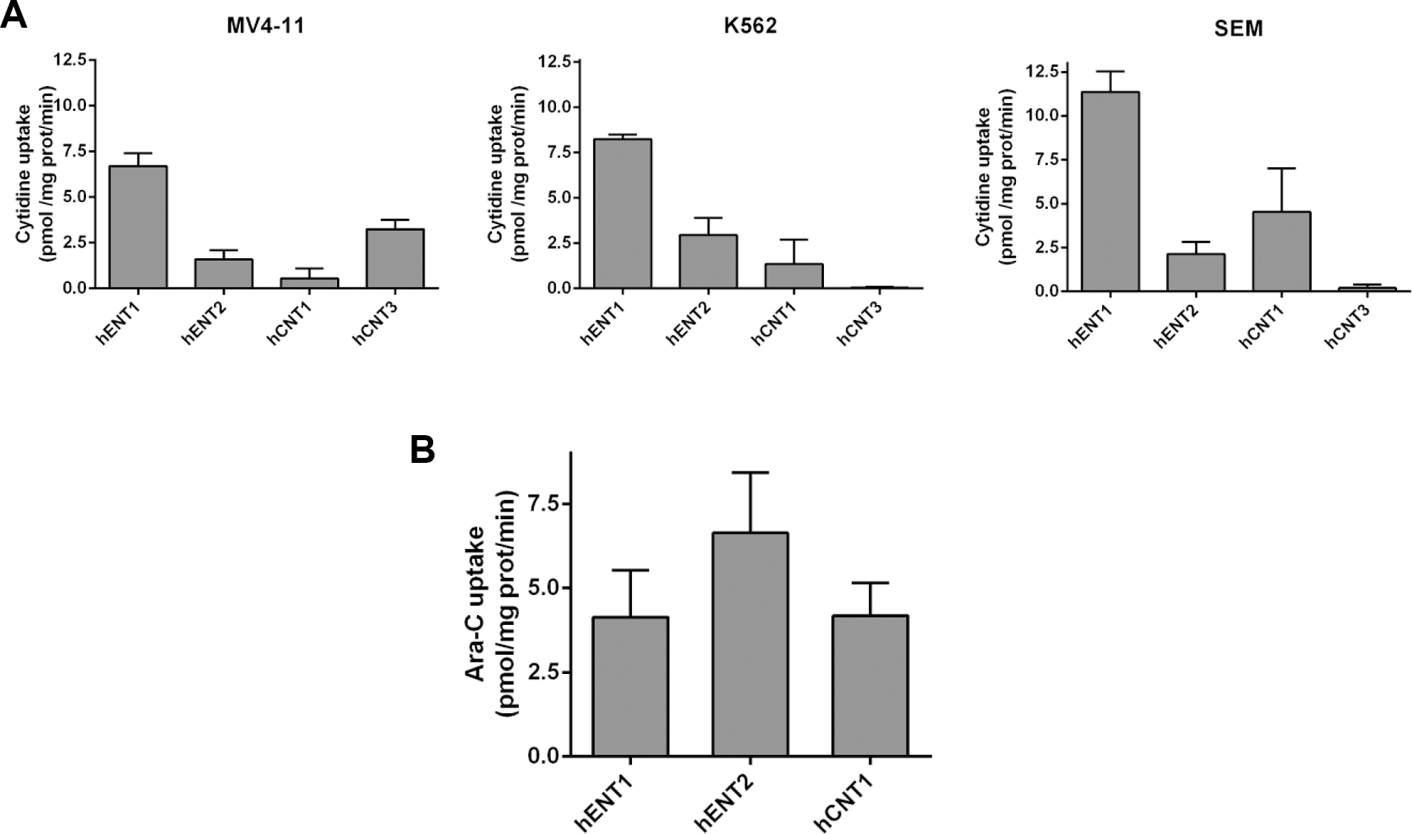
Characterization of nucleoside transporter activity implicated in Ara-C uptake (**A**) [^3^H]-cytidine uptake (1 μM, 1 min) by MV4-11, K562 and SEM cells and (**B**) [^3^H]-Ara-C transport (1 μM, 1min) in MV4-11 cell line were assayed. Cross-inhibitions were performed by adding to the transport medium cold guanosine (100 μM) and inhibition of hENT1 was achieved adding NBTI (1 μM) to the transport medium. Data are expressed as means ± SEM of triplicate measurements from three independent cultures.

Knowing that all cell lines expressed almost all NT mRNAs, we then aimed at determining the transportability profiles for each cell line. For this purpose, we performed radiolabeled cytidine uptake measurements. We chose cytidine because it is a pyrimidine that it is known to be a good substrate for hENTs, hCNT1 and hCNT3, all the putative Ara-C transporters. All cell lines showed hENT1-related function (Figure 2A) which was found to be the predominant nucleoside transporter activity in the three cell lines. We also detected, albeit to a lesser extent, hENT2 and hCNT1-related activities in all of them. MV4- 11 also retained some hCNT3-related function, being this activity undetectable in K562 and SEM cells.

### Nucleoside transporters implicated in Ara-C uptake

Direct transport of Ara-C by each of the previously identified NTs was determined. Given that the only cell line that retained all the NT-related activities was MV4- 11, we decided to focus on this cell line to study Ara-C transport. We observed that Ara-C was translocated inside cells by hENT1, hENT2 and hCNT1 (Figure 2B).

### FLT3 effect on hENT1 expression and activity

**Figure 3:**
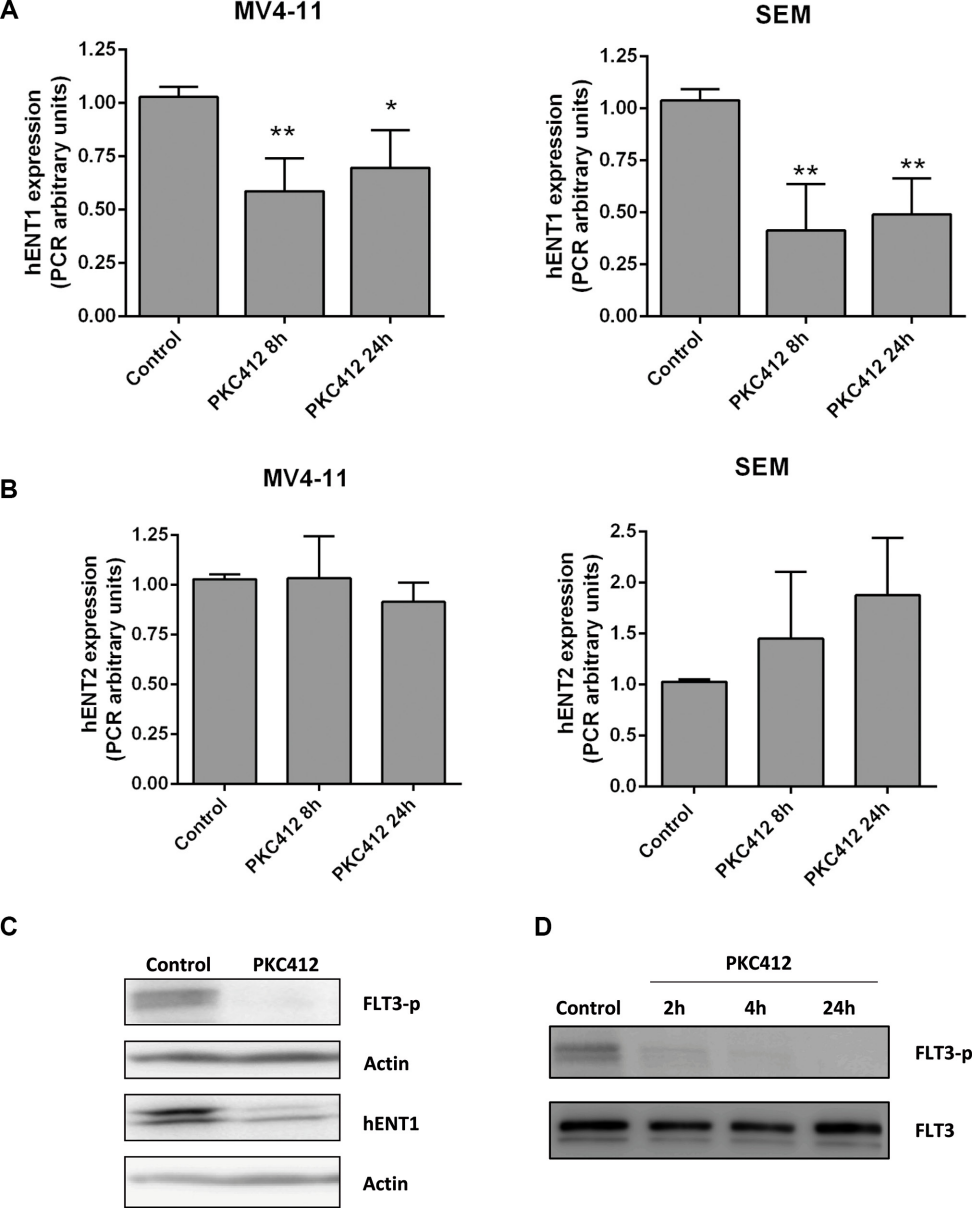
Involvement of FLT3 in *hENT1* expression mRNA expression of (**A**) *hENT1* and (**B**) *hENT2* were assayed by quantitative RQ-PCR in MV4-11 and SEM cells treated for 8 or 24 h with FLT3 inhibitor PKC412 (0.045 μM). Results are based on duplicate measurements from three independent experiments. Western blot for hENT1 (**C**), FLT3 and phospho-FLT3 (Tyr591) (**D**) were performed in cell extracts from MV4-11 cells incubated in the presence of PKC412 (0.045 μM) for different periods of time. A representative Western blot of three independent experiments is shown in each panel. Statistical significance denotes significant difference relative to control cells (*p < 0.05; **p < 0.01).

As we previously detected a positive correlation between *FLT3* and *hENT1* expression in the cohort of primary samples from pediatric patients we decided to evaluate the role FLT3 plays in modulating hENT1 expression. For this purpose we initially analyzed the effects of the FLT3 inhibitor, PKC412, on MV4-11 and SEM cell lines. All NTs and ME mRNA levels were determined in MV4-11 and SEM cells either in the presence or in the absence of PKC412 used at a concentration based upon previous literature [20]. Acute treatment with PKC412 resulted in a significant down-regulation of *hENT1-*related mRNA levels at the first studied time-point (8 h) without any further change at 24 h after treatment (Figure 3A). No changes in *hENT2*-related mRNA levels were found under the same conditions (Figure 3B). The down-regulation of the hENT1-related mRNA levels resulted in decreased hENT1 protein amounts (Figure 3C). Overall these data suggest that down-regulation of hENT1 is somehow specific and occurs immediately after PKC412 treatment no matter whether activated FLT3 is the result of its overexpression (SEM cells) or the consequence of mutations resulting in the constitutive activation of this kinase (MV4-11). Under the experimental conditions described above, it was also demonstrated that PKC412 inhibited FLT3 phosphorylation, without affecting total FLT3 expression (Figure 3D) proving that the inhibitor was acting as expected. To further assess the involvement of FLT3 in hENT1 regulation, another FLT3 inhibitor likely to be more specific than PKC412, AC220, was also used. AC220 could also inhibit FLT3 phosphorylation, thereby inducing a significant down-regulation of hENT1 protein expression (Supplementary Figure S2).

The functional impact of hENT1 down-regulation following FLT3 inhibition was demonstrated by monitoring hENT1-mediated cytidine uptake in MV4- 11 cells. hENT1 function was significantly decreased reaching its maximum inhibition at 16 h incubation with PKC412 inhibitor (Supplementary Figure S3).

### Effect of FLT3 inhibition on Ara-C uptake and cytotoxicity

**Figure 4:**
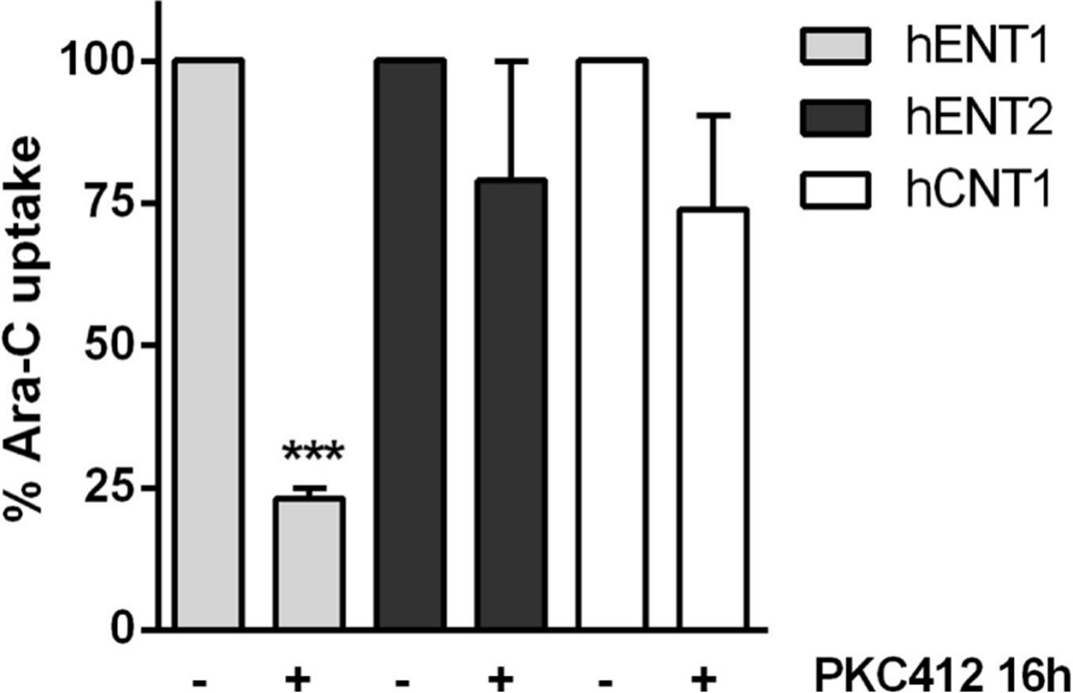
Effect of FLT3 inhibition in the uptake of Ara-C Direct uptake of [^3^H]Ara-C (1 μM, 1 min) was measured in MV4-11 cells in the presence of PKC412 for 16 h either in medium containing NaCl or choline chloride. Sodium-dependent transport was calculated as uptake in NaCl medium minus uptake in choline chloride medium. Data are normalized to uptake measured in the absence of PKC412 and expressed as percentage ± SEM from 3 independent experiments, each conducted in quadruplicate. Statistical significance denotes significant difference relative to control cells (***p < 0.001).

Considering that FLT3 inhibition induced the down-regulation of *hENT1* mRNA expression and related activity, without significantly affecting other NTs, we then aimed at determining the effect of FLT3 inhibition on Ara-C transport. For this purpose, we cultured MV4- 11 cells with PKC412 for 16 h and evaluated direct Ara-C uptake mediated by the previously demonstrated entities that were responsible for the transport of the drug, hENT1, hENT2 and hCNT1. Under conditions of maximal FLT3 inhibition, hENT1-mediated Ara-C uptake was dramatically decreased (Figure 4), without significantly affecting hENT2 and hCNT1 related activities. However, the residual hENT1 activity was still significant, representing nearly a 25% of that of untreated cells. In any case, no compensatory up-regulation associated with the other Ara-C transporters was observed.

**Figure 5:**
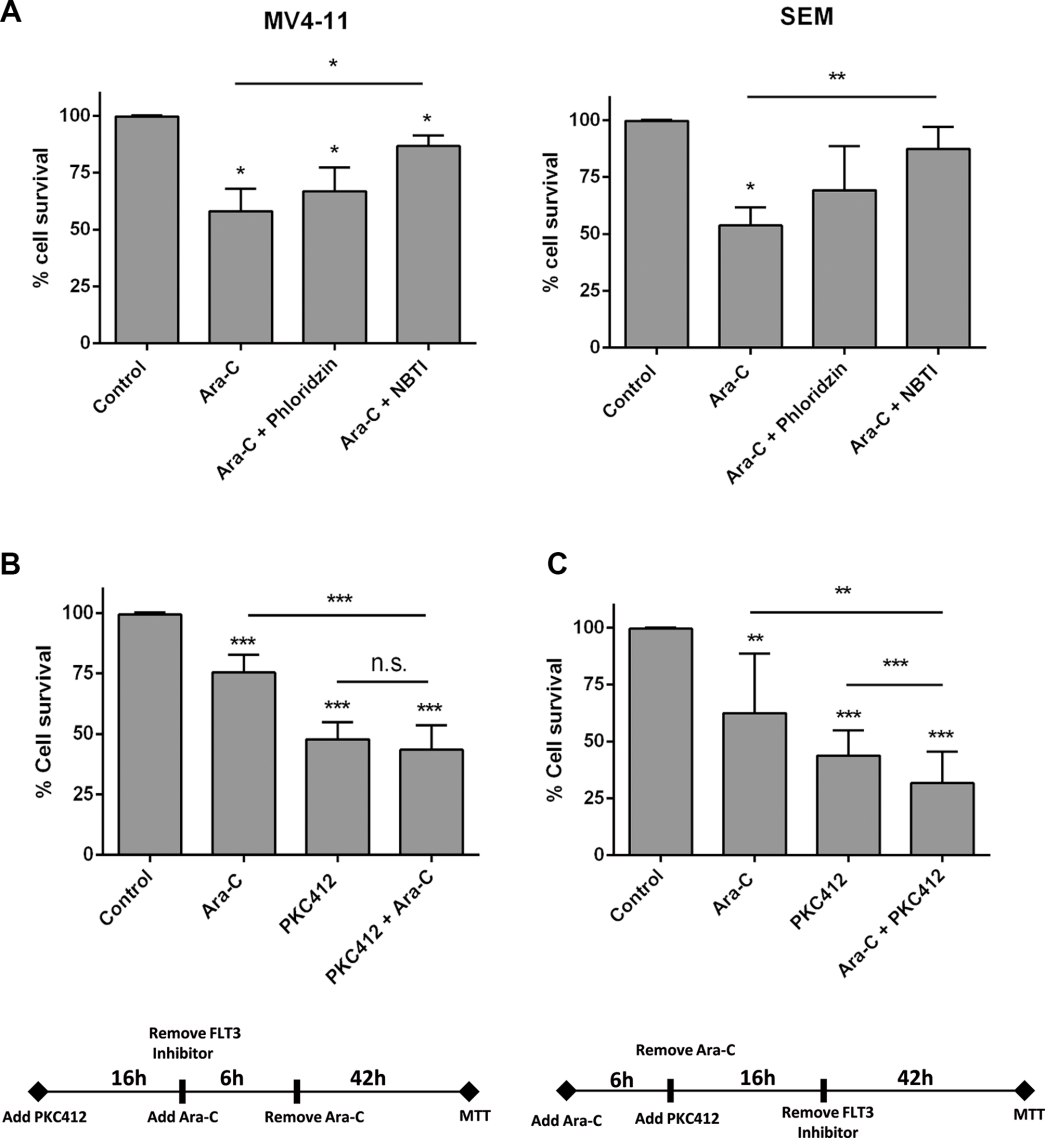
Effect of FLT3 inhibition in the cytotoxicity induced by Ara-C Cell viability was determined by MTT assays when (**A**) MV4-11 and SEM cells were cultured with Ara-C (3 μM and 1 μM for MV4-11 and SEM cells respectively) either in the presence or not of NBTI (1 μM) or phloridzin (250 μM) for 48 h; (**B**) MV4-11 cells were treated with the FLT3 inhibitor PCK412 (16 h), followed by a 6 h exposure to Ara-C (10 μM), and (**C**) MV4-11 cells were cultured with Ara-C (10 μM) for 6 h, followed by a 16 h exposure to PKC412. Data are expressed as percentage of survival ± SEM of triplicate measurements from six-nine independent experiments. Statistical significance denotes significant difference relative to control cells (*p < 0.1; **p < 0.01; ***p < 0.001) or to Ara-C or PKC412 treated cells as indicated.

To address the question of whether Ara-C cytotoxicity could be affected by hENT1 down-regulation under FLT3 inhibition conditions, cytotoxicity assays were performed. Ara-C was used at IC_50_ concentrations in SEM and MV4-11 cells (not shown). Under conditions of hENT1 activity inhibition (using 1 μM NBTI) Ara-C induced cytotoxicity was significantly blocked, whereas addition of phloridzin (an hCNT inhibitor) did not significantly alter Ara-C action (Figure 5A). Thus, in basal conditions (no PKC412 present), Ara-C induced cytotoxicity was mostly related to hENT1 function. When MV4-11 cells were treated with the FLT3 inhibitor PKC412 for 16 h, followed by a 6 h exposure to Ara-C, impact on cell survival was not significantly greater than when treating the cells with PKC412 alone (Figure 5B); however, if the exposure to the drugs was the opposite (6 h Ara-C treatment, followed by a 16 h exposure to PKC412), this combined treatment resulted in a significantly greater cell death than when treating the cells with PKC412 alone (Figure 5C). Thus, a lower cytotoxicity was observed if PKC412 was given previous to Ara-C exposure, probably due to a reduction in hENT1 activity. In contrast, once Ara-C had been administered, the subsequent addition of PKC412 resulted in greater cytotoxicity. In the former experimental design, the calculation of the coefficient of drug interaction (CDI) revealed the occurrence of drug antagonism (CDI: 1.20), whereas in the latter design, the effect of both drugs turned out to be additive (CDI: 1.05). Interestingly, when these experiments were performed using the FLT3 inhibitor AC220, similar results were obtained (Supplementary Figure S4), thereby reinforcing the key role of FLT3 in hENT1 modulation and cytarabine-associated cytotoxicity.

## DISCUSSION

*FLT3* mutations have emerged in the last years as key prognostic biomarkers in AML. FLT3-ITD mutation is a recognized adverse prognostic factor for outcome in adult and pediatric AML patients [2, 3, 5, 7, 8, 21–23]. Accordingly, patients harboring FLT3- ITD mutations are considered as high-risk patients, and benefit from hematopoietic stem cell transplant (HSCT) in first complete remission (CR1) [2, 21, 24]. *FLT3* overexpression is associated with FLT3 phosphorylation, thereby resulting in constitutive activation of the receptor, similarly to FLT3 mutants [3, 5, 8, 12]. Some authors have correlated *FLT3* overexpression, in absence of *FLT3* mutation, with poor outcome in both ALL and AML [4, 5, 8]. Beyond these clinical observations, little is known about the exact biological mechanism that confers high risk in such patients. Armstrong and co-authors [25] revealed that *FLT3* gene overexpression is the most distinctive feature of *MLL* rearranged acute leukemia. Moreover, a significantly higher *FLT3* expression level was observed in infant ALL-MLL+ patients. Interestingly, infant patients are highly sensitive to Ara-C, but show *in vitro* resistance to prednisone and asparaginase [1]. All these data have been taken into account to design the chemotherapeutic schemes in the International Cooperative Treatment Protocol for Infants with ALL, INTERFANT (99 & 06) [26].

Ara-C is mainly, but not exclusively, transported by hENT1, being this plasma membrane transporter a major contributor to Ara-C bioavailability and, likely, action. In fact, high *hENT1-*related mRNA levels have been reported in ALL-MLL+ patients, and this evidence may explain, at least partially, their Ara-C sensitivity [17]. The same group demonstrated that *MLL* rearrangements were no directly involved in Ara-C sensitivity in infant ALL and childhood AML-MLL+ [30], pointing out that other mechanisms are involved in Ara-C sensitivity in these patients.

In our study, the analysis of a cohort of 50 pediatric leukemia patients revealed a positive correlation between the expression (mRNA levels) of *FLT3* and *hENT1*. These results would be consistent with previous observations showing that subgroups of patients with high sensitivity to cytarabine frequently overexpress FLT3 [3, 10, 27]. Our results showing high *hENT1* and *FLT3* expression and a positive correlation between both parameters suggest that FLT3 might play a relevant role on Ara-C sensitivity, likely to involve hENT1 up-regulation.

We could not find any significant association between *FLT3* expression levels and other clinical or biological features, although this analysis was far out of the scope of our study. Moreover, a bias related to the selection of determined molecular subtypes in our cohort of patients was also introduced during the design of our study.

mRNA levels of other putatives Ara-C transporters (*hENT2, hCNT1, hCNT3*) and those of the intracellular metabolizing enzymes *DCK* and *cN-II* were also determined in the same cohort of patients, although no correlations among NT and ME and clinical and biological parameters were observed. Previous studies have suggested a correlation between specific NT expression profiles and outcome in solid tumors and hematological malignancies [15, 19, 28, 29], being this issue recently reviewed by ourselves [14]. However, the limited number of patients within the present cohort did not allow us to establish prognostic correlations.

We also found a positive correlation between *FLT3* and *DCK* expression (Supplementary Figure S1), the kinase involved in the first phosphorylation step of nucleoside analogs once they have entered the cells via NTs. Low levels of DCK tend to correlate with Ara-C resistance in childhood ALL and AML and adult AML [18, 30, 31]. Our observations suggest that DCK would not be a limiting step in Ara-CTP formation in patients with high FLT3 expression and thus, would support intracellular activation (i.e. phosphorylation) of Ara-C. These results would be in agreement with other studies [19] showing an *in vitro* correlation between hENT1 and DCK expression and nucleoside analogs sensitivity in pediatric and adult AML [18]. Recently, a collaborative EORTC-GIMEMA trial has demonstrated a better response rate and survival with high dose Ara-C in very high risk setting patients, including AML FLT3-ITD cases [22], that presumably would show high hENT1 expression according to our results.

Nucleoside analogues are the mainstay treatment in AML and are broadly used in relapsed-refractory ALL salvage schedules with diverse success rate [32–36]. As we here demonstrate a significant correlation between FLT3 and Ara-C transporter hENT1, we propose that FLT3 expression could be used as a nucleoside analogue sensitivity surrogate.

Not surprisingly, several FLT3 inhibitors have been developed in the last few years, and some of them are currently used in advanced-phases clinical trials with variable success rate (https://clinicaltrials.gov/). FLT3 inhibitors are basically expected to inhibit cellular proliferation through the down-regulation of this kinase, thus resulting in a withdrawal of antiapoptotic signals [37–39]. Some FLT3 inhibitors revealed higher efficacy when combined with chemotherapy [38, 40–42]. However, the best schedule combination with conventional chemotherapy has not been identified yet and research using different approaches to overcome inhibitors resistance is still needed. Given that a significant correlation was found in our study between *FLT3* expression and *hENT*1 expression, we also wanted to explore how FLT3 inhibitors could modulate Ara-C uptake and related cytotoxicity. We verified that Ara-C is transported by hENT1 as it was previously reported, and we also found that Ara-C can also be transported into the cells by hENT2 and hCNT1. We next demonstrated that the FLT3 inhibitor PKC412 induced a significant reduction of *hENT1* mRNA expression in both MV4-11 and SEM cell lines, which results in functional down-regulation of hENT1-dependent drug uptake. Similar effects on hENT1 down-regulation were found with AC220, a more selective FLT3 inhibitor. This effect appears to be specific, as PKC412 does not modify other transporter functions (i.e. hENT2 and hCNT1). Taken together, our results suggest that Ara-C transporter hENT1 expression and activity are regulated by FLT3. Interestingly, Jin and colleagues [20], using heterologous transduction of FLT3- ITD in HF6 cells, had suggested that FLT3-ITD down-regulates hENT1 expression in a heterologous system using transduced FLT3-ITD in HF6 cells. Oppositely, our results achieved in endogenously expressing FLT3 cell lines (wild type FLT3 and FLT3-ITD) suggest that FLT3 is indeed up-regulating *hENT1* expression and related activity. Those results are fully consistent with the clinical data, showing a positive correlation between *FLT3* and *hENT1* mRNA expression levels in the cohort of 50 patients here analyzed.

Regarding the effect on cell survival of the combination of a FLT3 inhibitor and a chemotherapeutic drug, two studies [43, 44] have previously demonstrated *in vitro* that combined therapies involving FLT3 inhibitors and chemotherapeutical agents might yield variable efficacy depending upon the sequence of drug treatment. Thus, antagonistic cytotoxic effects were induced when they pretreated FLT3-ITD cell lines and primary patient samples with a FLT3 inhibitor followed by chemotherapy. Similar results were obtained in wild type FLT3 with high *FLT3* mRNA expression levels. This effect appears to be the result of the FLT3 inhibitor inducing cell cycle arrest at the G1-S phase, thereby perturbing the action of genotoxic drugs, such as Ara-C. The possibility of FLT3 modulating hENT1 as a result of cell cycle perturbation should not be ruled out, although it looks unlikely to us. hENT1 has been shown to provide extracellular nucleosides for DNA synthesis in murine primary bone marrow macrophages [45], but hCNT1 is the transporter known to be cell cycle-dependent, showing up-regulation at the S phase [46]. Moreover, whereas FLT3 inhibition induces down-regulation of hENT1 it does not change at all either *hCNT1* mRNA expression levels or hCNT1-related activity. In summary, we think FLT3-related hENT1 modulation might not be the indirect result of cell cycle arrest induced by FLT3 inhibition. However, our data do further support the relevance of the sequence of drug treatment. In fact, under conditions of hENT1 down-regulation, the cytotoxic effect triggered by either PKC412 or AC220 was not further potentiated by Ara-C treatment, whereas this was not the case when cells were first treated with Ara-C, and basal hENT1 activity had not been yet down-regulated by FLT3 inhibition. Under these conditions the cytotoxic efficacy of the combined therapy was significantly much greater than when treating the cells with the FLT3 inhibitor alone. This finding highlights also the need for a better understanding of the basal regulatory properties of drug transporters, which may be targets of kinase inhibitors used in combined therapies.

In summary, we have demonstrated that Ara-C transport into cells is mediated by hENT1, hENT2 and hCNT1. More importantly, we also demonstrated that FLT3 significantly regulates hENT1 expression and activity and thereby Ara-C sensitivity. We have also proved that the sequence of the administration of FLT3 inhibitors and Ara-C is important, as the cytotoxic efficacy of the latter is greater when FLT3 inhibitors follow Ara-C administration. Overall, our data might contribute to better understand how FLT3 may influence drug sensitivity and to develop new therapeutic approaches in order to improve the overall outcome of patients with high risk subtypes of leukemia.

## MATERIALS AND METHODS

### Ethics statement

Investigation has been conducted in accordance with the ethical standards and according to the Declaration of Helsinki and according to national and international guidelines and has been approved by the authors' institutional review board.

### Patient samples and cell lines

Among 265 pediatric patients aged 0–18 years diagnosed with acute leukemia from 2003 to 2013 in Hospital Sant Joan de Déu, we selected 50 cases (B-cell precursor ALL: 44; T-ALL: 2; AML: 4) with available biological samples for the study. We selected cases with the main genetic abnormalities reported in ALL but, as we wanted to determine the possible influence of FLT3 over nucleoside transport activity, we conducted a bias towards cases with expected high *FLT3* expression, that is, patients with ALL and hyperdyploidy (51-67 chromosomes, HeH) and *MLL* rearranged cases (ALL-MLL+). The main clinical and biological characteristics of patients are described in Table 1. Patients were all uniformly treated according to the Spanish Society of Pediatric Hematology and Oncology (SEHOP) consecutive protocols SHOP-LAL-99 & 05 (ALL cases) and SHOP-LAM-00 & 07 (AML cases). We used normal bone marrow CD34+ cells as calibrator for *FLT3* and *hNT* expression. According to the Local Ethics Committee of our institution, all samples were stored in the legally competent Biobank of our Hospital and were used after informed consent was obtained either from the patients or their legal tutors.

**Table 1:** Clinical and biological characteristics of the 50 patients diagnosed with acute leukemia included in our study

	*N = 50*
Age, years (range)	4.3 (0–16)
Gender, n (%)	
Male	26 (52)
Female	24 (48)
CNS, n (%)	
CNS1	46 (92)
CNS2t^a^	3 (6)
CNS3	1 (2)
WBC count, ×10^9^/L, median (range)	17.6 (1.1–331.2)
Hemoglobin, g/L, median (range)	7.7 (2.9–11.7)
Platelets, ×10^9^/L, median (range)	52 (2–520)
Blasts, median (range)	
Bone marrow	93 (58–100)
Peripheral blood	55 (0–99)
Immunophenotype, n (%)	
Precursor B-ALL	44 (88)
T-ALL^b^	2 (4)
AML	4 (8)
Genetics, n (%)	
ALL:	
*HeH*	19 (38)
Other^c^	16 (32)
*ETV6-RUNX1*	4 (8)
*MLL+*	3 (6)
*TCF3-PBX1*	2 (4)
*BCR-ABL1*	2 (4)
AML:	
*MLL+*	3 (6)
Other^d^	1 (2)

a CNS2t: traumatic lumbar puncture.

b Two cases of Early T-cell Precursor T-ALL, one of them harboring a FLT3-ITD mutation, were included.

c Other B-cell precursor ALL cases (n = 14) included patients with normal karyotype (n = 5), cases with < 20 assessable metaphases (n = 6), cases with abnormalities at chromosome 9p (n = 3). The two T-cell ALL cases included had a normal karyotype.

d Other AML cases: we included a patient with 47, XX, t(6;9)(p23;q34), + 13[6]/46, XX, t(6;9)(p23;q34)[6] harboring a FLT3-ITD mutation.

Note: CNS: central nervous system. WBC: white blood cell. PB: peripheral blood. ALL: acute lymphoblastic leukemia. AML: acute myeloblastic leukemia. HeH: high hyperdiploid ALL (51–67 chromosomes).

We analyzed 3 different acute leukemia cell lines, SEM (childhood B-cell precursor ALL with translocation t(4;11) and *MLL* rearrangement with demonstrated high *FLT3* expression and no *FLT3* mutation; DSMZ ACC 546); MV4-11 (childhood AML with translocation t(4;11) and *MLL* rearrangement, harboring a FLT3-ITD mutation; DSMZ ACC 102) and K562 (lymphoid blast crisis of chronic myeloid leukemia with known low *FLT3* expression and no *FLT3* mutations; DSMZ ACC 10). K562 and MV4-11 cells were routinely cultured in RPMI-1640 medium (Lonza, Walkersville, MD) supplemented with 10% heat-inactivated Fetal Bovine Serum (FBS) (Life Technologies, Carlsbad, CA), 50 μg/ml penicillin-streptomycin (Invitrogen, Carlsbad, CA), and 2 mM L-glutamine (Invitrogen, Carlsbad, CA). SEM cells were cultured in Iscove's Modified Dubecco's Medium (IMDM) (Gibco, Breda, Netherlands) similarly supplemented with 10% heat-inactivated FBS, 50 μg/ml penicillin-streptomycin and 2 mM L-glutamine.

### DNA and RNA extraction and reverse transcription

Bone marrow or peripheral blood samples from diagnosis were used for the analysis. Separation of mononuclear cells was performed using density gradient with Ficoll-Hypaque (Sigma, St Louis MO, USA) and DNA was extracted with Qiaquick DNA extraction kit (Qiagen, Hilden, Germany). Total RNA was extracted with TriPure (Roche Diagnostics, Indianapolis, IN) and cDNA synthesis was performed from 10 ng of total RNA, using the QuantiTect Whole Transcriptome Kit (Qiagen, Hilden, Germany).

### FLT3 mutational status

FLT3-ITD was studied by amplification of the juxtamembrane domain spanning exons 14 and 15, using fluorescently-labeled primers and subsequent analysis on a 3130XL Genetic Analyzer (Applied Biosystems, Life Technologies, Foster City, CA, USA), as previously described [47]. To detect point mutations in codon D835 or deletions within codon I836, we amplified the exon 20 of *FLT3* and digested the product with Eco-RV enzyme, as reported [48]. All positive cases were directly sequenced to confirm the presence of mutations, using the BigDye Terminator Cycle Sequencing Kit v3.0 (Applied Biosystems, Life Technologies).

### Quantification of FLT3 mRNA and protein expression

The quantification of the *FLT3* gene-related mRNA was performed by real-time quantitative PCR (RQ-PCR) using the TaqMan^®^ Gene Expression Assay Hs00174690_m1 (Applied Biosystems, Life Technologies) (see Supplementary Table S2), in a Light-Cycler 480 II (Roche Diagnostics, Indianapolis, IN), according to the manufacturer's instructions. Relative quantification was calculated with the 2^−ΔΔCt^ method, using β-glucuronidase (*GUS*) (NM_000181.2, ref. 4310888E) as endogenous gene and normal bone marrow CD34+ cells as calibrator.

FLT3 protein levels before and after treatment with the FLT3 inhibitors PKC412 (Enzo Life Science, Plymouth Meeting, PA) and AC220 (Selleck Chemicals, Houston, TX) were quantified by Western blot. For this purpose, cells were treated with either PKC412 (0.045 μM) or AC220 (0.5 nM) and lysed in a buffer containing 20 nM Tris-HCl, 150 mM NaCl, 1 mM EDTA, 1% triton X-100 supplemented with 1 mM sodium orthovanadate, protease inhibitor (Complete mini; Roche, Basel, Switzerland) and phosphatase inhibitor cocktails (PhosSTOP; Roche). Fifty μg of proteins were separated by SDS-PAGE on standard 10% gel and transferred to Immobilon-P membranes (Millipore, Berford, MA, USA). Membranes were incubated with FLT3 (8F2) and phospo-FLT3 (Tyr591) antibodies (Cell Signalling, MA, USA). After washing with TBS-Tween, membranes were incubated with horseradish peroxidase (HRP)-conjugated secondary antibodies. Immunoreactive bands were detected by chemiluminiscence (ECL, Amersham Pharmacia Biotech, NJ, USA).

### Quantification of NT and metabolizing enzymes

Real-time quantitative PCR (RQ-PCR) amplification of hCNTs and hENTs were performed with primers and probes from Applied Biosystems, summarized in Supplementary Table S2, using the TaqMan Universal Master Mix, 700 nmol/L probe and 150 nmol/L of each primer in the ABI Prism 7700 Sequence Detection System (Applied Biosystems). The relative mRNA level of each gene was calculated with the 2^−ΔΔCt^ method and normalized to that of β-glucuronidase (*GUS*) expression level and normal bone marrow CD34+ cells as calibrator. Absolute RQ-PCR results were obtained from interpolation of ΔCT of each sample in line regression standards for number of cDNA copies of each gene. hENT1 protein levels were semi-quantified by Western Blot, using a commercial anti-hENT1 polyclonal antibody (STJ96396) from Saint John's Laboratory Ltd. (London, UK).

### hNT activity assays

Nucleoside uptake was measured in MV4-11, SEM and K562 cells using a method adapted from a technique previously described by our laboratory [49]. Cells were washed twice and resuspended in either a 137 mM NaCl or 137 mM choline chloride buffer. Uptake assays were started by mixing cell suspensions with a 10% of the final volume of the same buffer, supplemented with a radionucleoside - [^3^H]Ara-C or [^3^H]cytidine – at a final concentration of 1 μM (specific activity 1 μCi/nmol) at a specific activity of 4000 dpm/pmol. Incubation was stopped after 1min (linear initial velocity conditions) by washing the cells three times in 1 mL of a cold buffer composed of 137 mM NaCl and 10 mM Tris (hydroxymethyl) aminomethane-HEPES (pH 7.4). Cells were then dissolved in 1 ml Triton-X-100 and aliquots were sampled for protein determination, according to Bradford (Bio-Rad, Hercules, CA), and for radioactivity measurements.

In the presence of Na^+^, hENTs and hCNTs are functional, although only hCNTs require this ion for substrate translocation. Thus, sodium dependent nucleoside transport activity (hCNT-related) was determined by subtracting uptake rates measured in the choline chloride medium (almost exclusively related to hENT1 and hENT2 activities) from measurements obtained in the sodium containing buffer (in which both ENT and CNT are active).

Cross-inhibition experiments were performed as described above, but adding saturating concentrations (100 μM) of a secondary non-radiolabeled nucleoside to the incubation media, which will compete for the transporter and block the transport of radiolabeled substrate. By adding extra guanosine to [^3^H]cytidine uptake media, hCNT3 but not hCNT1 activity will be blocked. hCNT1-mediated uptake was determined by substracting the activity measured in [^3^H]cytidine + guanosine conditions from [^3^H]cytidine alone and hCNT3 related activity from the substraction of [^3^H]cytidine + guanosine transport activity from the one determined when using [^3^H]cytidine alone. The equilibrative (Na^+^-independent) transport component inhibited by NBTI (1 μM) accounts for the hENT1-related nucleoside transport activity, whereas the NBTI-resistant transport includes residual hENT2-related uptake plus diffusion and binding components, which in general are negligible.

### Cell treatment and apoptosis detection by MTT assay

For routine cytotoxicity assays, 2 × 10^4^ cells were cultured either in the presence or the absence of appropriate inhibitors. To unveil the role selected nucleoside transporters can play in cytarabine-induced cytotoxicity cells were incubated for 15 min either in the presence or in the absence of NBTI (1 μM for hENT1 inhibition) or phloridzin 250 μM (for hCNT1inhibition). Afterwards Ara-C was added (3 μM and 1 μM for MV4-11 and SEM cells respectively) and cytotoxicity determined using 3-(4,5-dimethylthiazol-2-yl)-2,5-diphenyltetrazoliumbromide (MTT; Sigma-Aldrich) after 48 h. Optical density (OD) was measured at 550 nm. For the analysis of combined drug effects cells were cultured with either 0.045 μM PKC412 or 0.5 nM AC220 and 10 μM Ara-C, following the experimental design described in the Results section, aimed at elucidating the probable role of the order of drug administration of chemotherapeutic efficacy. The Coefficient of Drug Interaction (CDI) was calculated as explained in [50].

### Statistical analysis

For the correlation of clinical and biological variables, we used the *χ*^2^ and the Fisher's exact tests for categorical variables and the Student's *t*-test or the Mann–Whitney *U*-test for non-parametric tests. The Spearman non-parametric test was used to determine correlations among mRNA expression values of the analyzed genes. In addition, a Kruskal–Wallis test was used to compare the gene expression levels of *FLT3* and NT among the different ALL subgroups. As we selected certain subtypes of leukemia according to their expected FLT3 expression and also the total number of primary cases is low, we did not perform survival analysis in our study. All *p* values were considered significant when < 0.05. All the statistical analyses were performed using the SPSS 22.0 statistical software package (SPSS Inc., Chicago, IL, USA).

## SUPPLEMENTARY MATERIALS FIGURES AND TABLES


