# Supplementary material for: FLT3 is implicated in cytarabine transport by human equilibrative nucleoside transporter 1 in pediatric acute leukemia
Source: Oncotarget. 2016 Jul 6;7(31):49786–99. doi: 10.18632/oncotarget.10448 (PMC5226548; doi:10.18632/oncotarget.10448)
Supplement: Supplementary file 1 [file oncotarget-07-49786-s001.pdf]

FLT3 is implicated in cytarabine transport by Human Equilibrative Nucleoside Transporter 1 in pediatric acute leukemia

Supplementary Materials

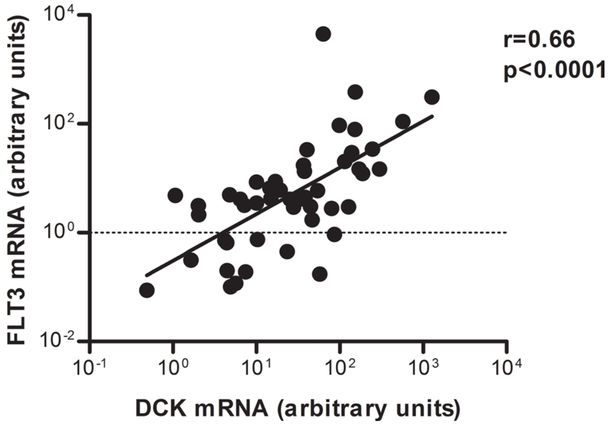

Supplementary Figure S1: Correlation between *DCK* and *FLT3* mRNA expression in pediatric leukemia samples. Relative *DCK* mRNA levels of cells from 50 pediatric patients with acute leukemia were plotted against the levels of *FLT3* mRNA in the same samples. Correlation coefficient and level of significance are shown in the figure.

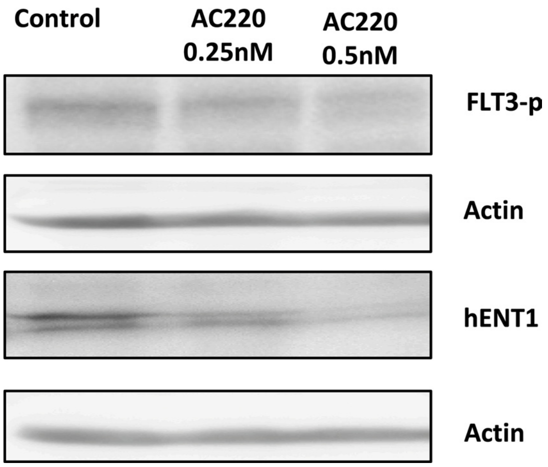

Supplementary Figure S2: Western blot analysis of hENT1 and phospho-FLT3 (Tyr591) were performed in cell extracts from MV4-11 cells. Cells were incubated in the presence of AC220 (0.25 nM and 0.5 nM) for 16 h. A representative Western blot out of three independent experiments is shown in each panel.

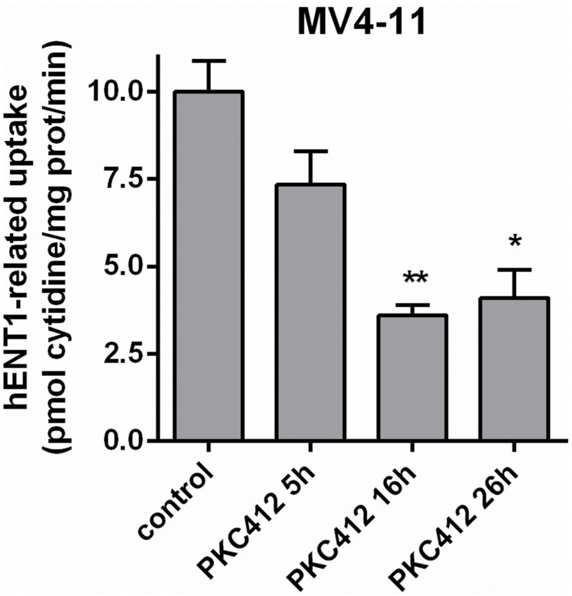

Supplementary Figure S3: Effect of FLT3 inhibition in hENT1-related activity. hENT1-mediated [^{3}H]cytidine uptake (1 µM, 1 min) was measured in MV4-11 cells in the presence of PKC412 for 5, 16 or 26 h. Mean ± SEM from 3 independent experiments, each conducted in quadruplicate, is shown. Statistical significance of the difference relative to control cells is as follows: $*p < 0.1$; $**p < 0.01$.

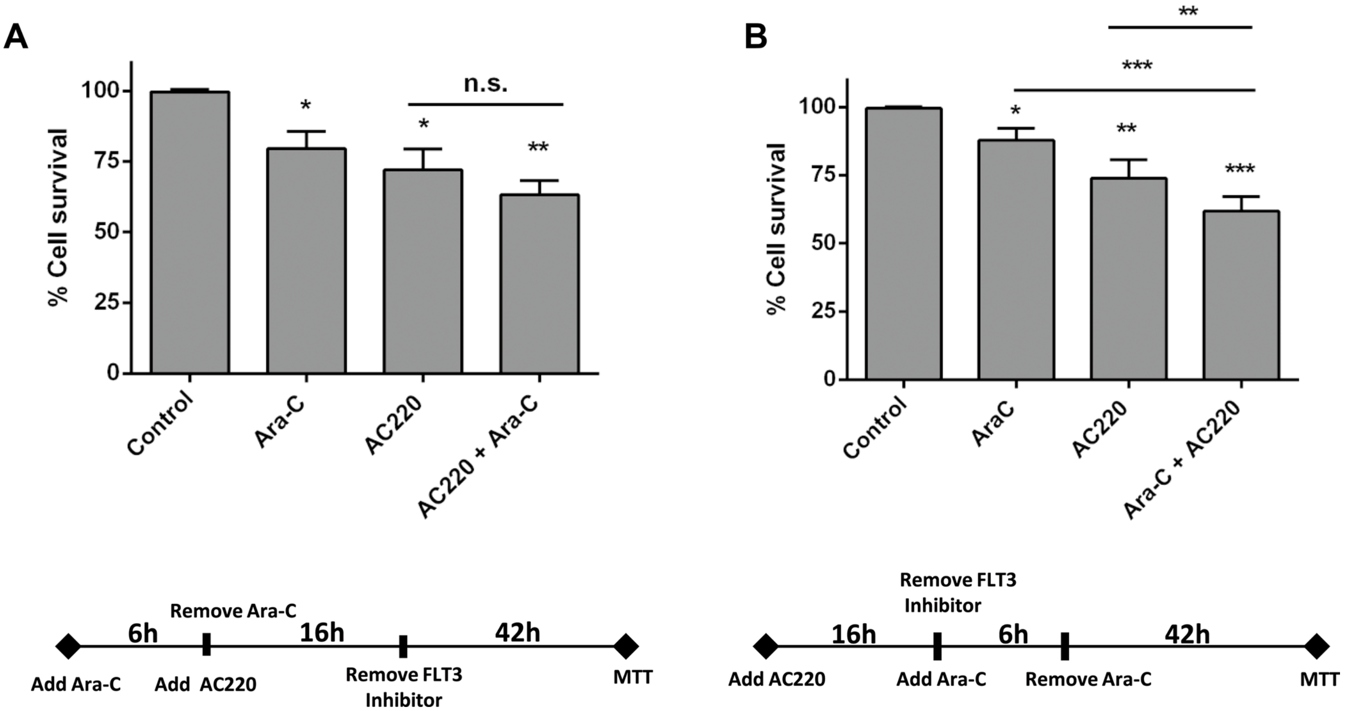

Supplementary Figure S4*:* Effect of AC220 on the cytotoxicity induced by Ara-C. Cell viability was determined by MTT assays when (**A**) MV4-11 cells were treated first with the FLT3 inhibitor AC220 for 16 h, then AC220 was removed from the medium and this was followed by a 6 h exposure to Ara-C (10 µM), and (**B**) MV4-11 cells were cultured first with Ara-C (10 µM) for 6 h, then AraC removed from the medium and followed by a 16 h exposure to AC220. Data are expressed as percentage of survival ± SEM of triplicate measurements from six independent experiments. Statistical significance of the differences relative to control cells is as follows: $*p < 0.1$; $**p < 0.01$; $***p < 0.001$. Interexperimental differences were similarly determined for Ara-C and PKC412 treated cells as indicated in the figures.

Supplementary Table S1: Absolute expression of hNTs under experimental conditions

RNA expression (log copies/µg RNA)			
hNT	**MV4-11**	**SEM**	**K562**
hCNT1 (SLC28A1)	3.04 ± 0.11	2.61 ± 0.05	2.82 ± 0.06
hCNT2 (SLC28A2)	2.88 ± 0.01	3.06 ± 0.04	2.69 ± 0.11
hCNT3 (SLC28A3)	5.06 ± 0.01	1.33 ± 0.01	1.69 ± 0.09
hENT1 (SLC29A1)	5.05 ± 0.10	5.30 ± 0.01	5.52 ± 0.17
hENT2 (SLC29A2)	2.55 ± 0.03	3.79 ± 0.06	4.17 ± 0.11

mRNA expression (log copies/µg RNA) quantified by absolute RQ-PCR.

Supplementary Table S2: Oligonucleotides and probes used in RQ-PCR to measure different nucleoside transporters, metabolizing enzymes and *FLT3* expression levels

target gene	encoded protein	sense 5-3/antisense 5-3′	probes 5´FAM-TAMRA3′
SLC28A1	hCNT1	TGATTTCTTGGAAAGCCTGGA/ TGCTCCTGATCTCTGCGG	AAGGCCAGCTCCCTAGGAGTGACTTGAG
SLC28A2	hCNT2	AAGTAGAGCCTGAGGGAAGCAA/ GCCCAGTCCATCCCCC	AGGACTGACGCACAAGGAACACAGCC
SLC28A3	hCNT3	GAGCTGTGCAAAGCAGGGA/ TGGCGAATCCTGCTCAACTGTG	CACACAAACACCAGGATGAAGAACAGG
SLC29A1	hENT1	GCAAAGGAGAGGAGCCAAGA/ TTCATTGGTGGGCTGAGAGT	CAGGCAAAGAGGAATCTGGAGTTTCAGTCTC
SLC29A2	hENT2	CCCTGGATCTTGACCTGGAG/ GGTTTTCCTGGCTTCTGGG	AGGAGCCGGAATCAGAGCCAGATGA
DCK	DCK	*Hs01040726_m1 (Applied Biosystems, Life Technologies)*	
FLT3	FLT3	*Hs00174690_m1 (Applied Biosystems, Life Technologies)*	
